# Will our dreams ever come true: treatment options for patients with HAM/TSP in 10 years

**DOI:** 10.1186/1742-4690-11-S1-O27

**Published:** 2014-01-07

**Authors:** Cynthia Pise-Masison

**Affiliations:** 1 Animal Models and Retroviral Vaccines Section, National Cancer Institute, Bethesda, MD, USA

## 

No abstract submitted.

